# HGF/Met Axis in Heart Function and Cardioprotection

**DOI:** 10.3390/biomedicines2040247

**Published:** 2014-10-21

**Authors:** Simona Gallo, Valentina Sala, Stefano Gatti, Tiziana Crepaldi

**Affiliations:** 1 Department of Oncology, University of Turin, Corso Massimo D’Azeglio 52, 10126 Turin, Italy; E-Mails: simona.gallo@unito.it (Si.G.); valentina.sala@unito.it (V.S.); stefano.gatti@unito.it (St.G.); 2 Department of Medical Sciences, University of Turin, Corso Dogliotti 14, 10126 Turin, Italy

**Keywords:** hepatocyte growth factor, Met tyrosine kinase receptor, heart development, cardiotoxicity, cardioprotection, myocardial infarction, angiogenesis, fibrosis, inflammation, cardiac regeneration

## Abstract

Hepatocyte growth factor (HGF) and its tyrosine kinase receptor (Met) play important roles in myocardial function both in physiological and pathological situations. In the developing heart, HGF influences cardiomyocyte proliferation and differentiation. In the adult, HGF/Met signaling controls heart homeostasis and prevents oxidative stress in normal cardiomyocytes. Thus, the possible cardiotoxicity of current Met-targeted anti-cancer therapies has to be taken in consideration. In the injured heart, HGF plays important roles in cardioprotection by promoting: (1) prosurvival (anti-apoptotic and anti-autophagic) effects in cardiomyocytes, (2) angiogenesis, (3) inhibition of fibrosis, (4) anti-inflammatory and immunomodulatory signals, and (5) regeneration through activation of cardiac stem cells. Furthermore, we discuss the putative role of elevated HGF as prognostic marker of severity in patients with cardiac diseases. Finally, we examine the potential of HGF-based molecules as new therapeutic tools for the treatment of cardiac diseases.

## 1. Introduction

Hepatocyte growth factor (HGF) is a pleiotropic cytokine controlling different cellular processes, such as migration, morphogenesis, apoptosis and proliferation. Molecularly, it is a large, multidomain protein, synthesized and produced as an inactive single chain pro-HGF precursor. The activation of HGF requires a proteolytic cleavage by proteases present in the serum or at the cell membrane. HGF mediates its biological functions through its unique tyrosine kinase receptor, Met. The latter is an αβ-disulphide linked heterodimer. The α-chain is completely extracellular. The β-chain contains an extracellular region, a single-pass transmembrane domain and the cytoplasmic portion of the receptor. The intracellular portion of the β-chain is composed of a juxtamembrane segment with regulatory function, the tyrosine kinase domain and the *C*-terminal region with the two tyrosines in the multifunctional docking site. Upon HGF binding, Met dimerizes and its tyrosine kinase activity is stimulated. Autophosphorylation of Met in the docking site recruits adaptor molecules and activates several intracellular signaling pathways, which include Akt/PI3K and MAPK cascades. The activation of Met-regulated signaling pathways starts a specific cellular program known as “invasive growth”. This process is induced in normal organ development, during repair of injured tissue and in disease.

In this review, we focus on the complex role of HGF/Met axis in the heart during physiologic and pathologic conditions. We initially describe its essential role in the maintenance of normal adult myocardial function. In this context, we provide our perspective on the cardiac implications of anti-cancer therapies that neutralize Met activity. Next, we analyze the cardioprotective actions mediated by HGF/Met after myocardial injury. Accordingly, we examine the protection against apoptosis and autophagy, the proangiogenic and anti-fibrotic action, the anti-inflammatory and immunomodulatory function and the ability of stimulating cardiac regeneration. Furthermore, we address the meaning of elevated HGF in the context of acuteness of cardiac diseases. Finally, we consider the potential therapeutic benefits of molecules which promote Met activity for the treatment of heart diseases.

## 2. The Role of Hepatocyte Growth Factor (HGF)/Met in Physiological Heart Development

Growth factors and Receptor Tyrosine Kinases (RTKs) have important roles in the morphogenesis and differentiation of the myocardium. Among growth factors, the HGF and its receptor (Met) have been implicated in cardiomyogenesis. Both *HGF* and *Met* receptor mRNAs are co-expressed in cardiomyocytes from E7.5, soon after the heart has been determined, to E9.5 [1]. Transcripts for HGF ligand and receptor are first detected before the occurrence of cardiac beating and looping and persist throughout the looping stage, when heart morphology begins to elaborate. Moreover, both *HGF* and *Met* mRNAs are detectable after the expression of the heart transcription factor Nkx2–5 and concomitantly with the cardiac actin gene. In avian studies, positive staining for HGF protein was found in the myocardial layer of the atrio-ventricular canal, in a stage of development in which the epithelial to mesenchymal transformation (EMT) of the endocardial cushion occurs [2]. These results suggest a role for HGF as one of the myocardial-derived factors capable of regulating some of the processes contributing to EMT. In the mouse, ablation of *c-Met* [3] or *HGF* [4,5] by homologous recombination results in embryonic lethality between days E12 to E14. These mice do develop a heart, indicating that HGF and Met are not essential for the initial heart development. The early lethality of these mice precludes the analysis of the heart in later developmental stages. However, conditional loss and gain of function models have been used to address the role of HGF/Met pair in heart development and function. Inactivation of the Met receptor in cardiomyocytes using the Cre-α-MHC mouse line has indicated that Met is dispensable for heart development [6]. In contrast, Met is required in adult mice to protect cardiomyocytes, by preventing age-related oxidative stress, apoptosis, fibrosis and cardiac dysfunction [6]. Transgenic mice with cardiac-specific tetracycline suppressible expression of either HGF or the constitutively activated Tpr–Met kinase also result in cardiac damage [7]. During the early postnatal period of rapid growth, neonatal cardiomyocytes express the Met receptor *in vivo* and can respond to exogenous HGF by activating PI3K/Akt, P38MAPK and Erk1,2 signaling and influencing both proliferating and differentiating parameters [7,8]. Expression of Tpr–Met in postnatal cardiomyocytes also leads to activation of both Akt and Erk1,2, thus, eliciting a growth signal. In terminally differentiated cardiomyocytes, this signal results in switching on a hypertrophic program, which, if prolonged, leads to heart failure [7]. Altogether, these results suggest that fine tuning of Met signaling is required for normal cardiac development and function. Notably, it has been recently found that mutations that affect components of the RAS–RAF–MEK pathway cause several developmental disorders, including Noonan, Costello and cardio-facio-cutaneous syndromes with many overlapping clinical symptoms (for a review, see [9]). Among other defects, patients present hypertrophic cardiomyopathy (HCM). Most of the genetic lesions encode proteins that belong to RAS pathway, indicating that hyperactivation of RAS signaling is involved in the pathogenesis of HCM and paving the way for the identification of new specific targets for the treatment of HCM.

## 3. The Potential Cardiotoxicity of HGF/Met Inhibitors

New anti-cancer therapies have been developed in the last ten years to target those RTKs whose continued expression has proved to be important for maintaining and driving cancer progression, a condition known as “oncogene addiction” [10]. The success of anti-HER2 and anti-VEGF receptor-targeted drugs has greatly encouraged the exploitation of therapies directed against other RTKs signaling pathways. However, it has appeared very soon that targeted therapies may have important side effects. Indeed, specific signaling pathways exert a function not only on cancer cells, but also on healthy tissues. In particular, the heart is vulnerable to the inhibition of those pathways which are targeted in cancer, as in the case of HER2-targeted therapy [11]. Thus, possible concerns about targeting signaling systems which are expressed in the heart and are known to play a role in cardiac development and response to stress must be paid attention to.

Cancer cells often display dysregulation of HGF/Met system, including autocrine and paracrine HGF production (and hence Met activation), and transcriptional overexpression or amplification of the *Met* gene. For this reason, Met-targeted cancer therapies have been developed and several HGF/Met inhibitors, including HGF neutralizing antibodies, Met down-regulating antibodies and Met Tyrosine Kinase Inhibitors (TKIs), are currently exploited in clinical trials [12]. Moreover, Met and HGF have been implicated in the acquired resistance to inhibitors of other RTKs, such as EGFR. Thus, combination therapies of Met and other RTK inhibitors have been taken in account as a novel powerful strategy to treat tumors with acquired resistance to TKI. Given the critical role played by HGF/Met couple in myocardial protection (see above and below), consideration must be given to the possible cardiac side effects of Met-targeted cancer therapy. Met inhibitors, such as Crizotinib or PF-04254644, have been tested by short-term treatments of cellular and pre-clinical models and have been shown to induce cardiomyocytes death through molecular mechanisms involving ROS production, activation of caspases, alteration of cell metabolism and blockage of ion channels [13,14]. Better understanding of long-term treatments and identification of the pathways which are mostly involved in the myocardial toxicity will enable the development of combinatorial therapies aimed at alleviating toxicity and allowing safer Met-based anti-cancer treatments. In conclusion, with respect to patients undergoing cancer therapy with HGF/Met inhibitors alone or in combination, the risk of cardiotoxicity is prominent and the evaluation of cardiac stressors in the design of the therapeutical approach is advisable.

## 4. The Cardioprotective Role of HGF/Met in Myocardial Infarction

HGF is accumulated in the ischemic myocardium both during permanent coronary artery occlusion [15] and in experimental ischemia and reperfusion (I/R) [16]. In human patients with acute myocardial infarction (MI), increased secretion of HGF in blood circulation is observed [17], probably as a consequence of myocardial damage. However, also humoral mediators, such as proinflammatory cytokines and neurotransmitters, might be responsible for the induced production of HGF in distinct organs, such as liver. Nakamura *et al.* [18] have demonstrated that HGF is required for self repair after MI: by blocking endogenous HGF activity with neutralizing antibodies, the infarct size worsened and cardiomyocyte death increased. Furthermore, when recombinant *HGF* or *HGF* gene transfer was administrated in the infarct area, cardiac function was ameliorated [18]. After this pivotal work, a great deal of evidence has shown the importance of HGF/Met system in cardiac repair after MI (for a review see [19]).

## 5. Anti-Apoptotic and Anti-Autophagic Function of HGF in Cardiomyocytes

Cardiomyocyte apoptosis is a key event for the pathogenesis of heart damage following I/R [20]. Supplementation with HGF counteracts oxidative stress in the setting of MI [18]. HGF protects cardiac cells against oxidative stress-induced apoptosis via activation of MEK/Erk1,2 [21], p38MAPK [6] and PI3K/Akt [22] pathway. Activation of Met signaling by HGF and agonist antibodies defends cardiac muscle cells against apoptosis, through inhibition of caspase activation [23,24]. Furthermore, HGF and Met agonist antibodies protect cardiac cells against hypoxic injury via the inhibition of autophagy [23]. Autophagy is a less renowned regulator of cell viability [25]. Under prolonged ischaemia, autophagy fails to be protective and becomes harmful, thus, contributing to cell death [23,26]. Mechanistically, mTOR is the crucial protective pathway downstream to Met acting against hypoxia-induced autophagic response [23]. Thus, the cardioprotective effects of HGF and Met agonist antibodies on cardiomyocytes are exerted by a dual mechanism resulting in enhanced cell survival in a hostile environment.

## 6. Proangiogenic Function of HGF toward Vascular Cells

HGF is a powerful angiogenic growth factor [27,28]. Its mitogenic effect in endothelial cells *in vitro* is stronger than that elicited by VEGF and bFGF [29]. HGF is also a potent stimulator of angiogenesis *in vivo* in hind limb ischemia models [30,31,32]. Exogenous HGF in the heart results in increased density of capillaries under MI [22,33,34], thus, suggesting that HGF/Met couple is essential to promote regeneration of endothelial cells and neovascularization during MI. Such enhanced angiogenesis contributes to protecting cardiomyocytes from ischemic injury [35]. In addition, HGF induces the release of other endothelial cell mitogens from non-endothelial cells [36,37,38,39], including VEGF. However, while VEGF causes endothelial permeability and oedema, HGF inhibits vascular permeability and inflammation [40], and also attenuates thrombin-induced endothelial permeability [41]. Moreover, the combination of VEGF and HGF has an additive effect on both proliferation and migration of endothelial cells and neovascularization *in vivo* [38]. In addition to neoangiogenesis, collateral vessels formation (arteriogenesis) has an important role in attenuating ischemic cardiomyopathy [42]. Maturation of blood vessels and arteriogenesis involve the activation of angiopoietin/Tie2 ligand receptor system and the communication between endothelial cells and smooth muscle cells (SMC). Notably, angiopoietin induces HGF and promotes the recruitment of SMC, enhancing the stabilization of the newly-formed blood vessels [43].

## 7. Anti-Fibrotic Action of HGF in Cardiac Fibroblasts

HGF is a powerful anti-fibrotic factor in the chronic injury of different organs, including liver, kidney and heart. HGF prevents fibrotic response in MI [34,44,45] and dilated cardiomyopathy [46,47]. Mechanistically, the anti-fibrotic action of HGF is mediated by the inhibition of TGF-β1 production [48]. Moreover, HGF mitigates TGF-β-initiated Smad signaling [49] and induces the expression of decorin, an inhibitor of TGF-β [50] in renal fibrosis. In lung fibrosis, HGF is also able to induce metalloproteases (MMP) [51], which promote ECM degradation and apoptosis of myofibroblasts.

The anti-fibrotic action of HGF occurs via a mechanism involving production of NO in doxorubicin-mediated cardiac damage [52]. Inhibition of NO production leads to the up-regulation of ACE [53] and accelerates fibrosis [54]. HGF increases NO in endothelial cells [55] and inhibits angiotensin II (ANG II) in the heart of cardiomyopathic hamsters [48], thus mitigating the fibrotic changes. Furthermore, ANG II and TGF-β are strong negative regulators of local HGF production [56,57]. Thus, decreased secretion of HGF by TGF-β and ANG II may result in the abnormal accumulation of ECM.

## 8. Anti-Inflammatory and Immunomodulatory Function of HGF

Acute inflammation is a common process during organ injury, contributing to tissue damage. It is initiated by cells that are already present in the tissue, mainly resident macrophages, dendritic cells and mastocytes, and by extravasation of leukocytes, mainly neutrophils, out of the blood stream into the tissue. HGF down-regulates the expression of adhesion molecules, such as ICAM-1/E-selectin, on endothelial cells, and therefore decreases the binding of leukocytes to the endothelium [58]. Monocytes are activated by HGF into macrophages [59], which release inflammatory mediators responsible for the clinical signs of inflammation. In macrophages, HGF down-regulates the production of IL-1, IL-6 and IL-18, via induction of heme oxygenase-1 (HO-1; [60]). Moreover, HGF up-regulates IL-4 and IL-10 in autoimmune myocarditis [61], indicating that HGF may also play a role in immunosuppression of T cell response.

Interestingly, HGF promotes the differentiation of macrophages into immunosuppressive dendritic cells, which favor the expansion of IL-10 producing regulatory T lymphocytes (T-reg), thus, maintaining T cells in a low state of activation [62]. This potent immunomodulatory mechanism of action of HGF has been also shown in an animal model of autoimmune encephalitis [63]. The exogenous administration of HGF up-regulates the serum levels of IL-10 and down-regulates those of IL-8 in patients with coronary heart disease [64]. IL-10 protects against atherosclerotic disease [65] by down-regulating the inflammatory process [66]. On the other hand, IL-8 acts as a proinflammatory cytokine with chemoattractant and mitogenic effects on different cells, including vascular smooth muscle cells [67] and monocytes [68]. Finally, the administration of HGF prolongs cardiac allograft survival by reducing IFNγ, a cytokine that triggers cardiac graft rejection [69]. IFNγ not only contributes to the inflammatory damage, but it also regulates T-reg [70]. These results suggest that the cardioprotective and immunomodulative properties of HGF in cardiac allografts and myocarditis might be at least in part ascribed to T-reg immune tolerance and T cell-mediated immunosuppression.

## 9. HGF and Cardiac Regeneration

Nowadays, it is generally accepted that the myocardium has regenerative capacity, though at extremely low extent. Mammalian heart renewal may occur either by pre-existing cardiomyocytes [71] or by cardiac progenitor cells (CPCs), which are still able to proliferate [72]. CPCs express Met, among other growth factors receptors [73]. Stimulation with exogenous HGF and IGF-1 promotes mobilization, expansion and differentiation of resident CPCs into cardiomyocytes and vascular cells. Various types of adult CPCs have been described based on specific surface markers and different isolation approaches [74]. CPCs may have different origins: some cells reside in the myocardium since fetal life [75], some others derive from bone marrow [76] and colonize the myocardium in the postnatal period [77,78]. CPCs have been injected into infarcted murine hearts, with the goal of triggering cardiac regeneration. This approach has provided a modest, albeit existing, improvement in cardiac function [77,78,79]. It is currently believed that the therapeutic benefit of transplanted stem cells is greatly due to their ability to secrete paracrine factors. Indeed, both resident and transplanted CPCs secrete HGF [80,81,82], enhancing the survival of cardiomyocytes to hypoxia, as well as inducing formation of new endothelium [83]. Thus, treatment of injured heart with a cocktail of growth factors, including HGF, may represent a valid strategy not only for cardioprotection but also for cardiac regeneration [82,84,85]. Recently, a population of cardiac progenitors of epicardial origin has been found, which promotes vessel formation and collateral growth [86] and contributes to cardiac repair after injury [87]. Interestingly, the pericardial fluid from patients with MI induces the activation of EMT in the epicardium [87]. Several factors initiating and/or controlling the EMT process during the embryonic development of the heart have been identified, including HGF [2]. Interestingly, increased HGF levels have been found in the pericardial fluid of ischemic patients [88] suggesting that HGF may contribute to the activation of EMT in the adult epicardium.

## 10. The Putative Role of Elevated HGF as Prognostic Marker of Severity in Patients with Cardiac Diseases

Although strong evidences demonstrate the beneficial role of HGF in the heart, different studies correlate enhanced HGF production with increased disease severity and mortality. Plasma of patients with advanced heart failure present increased levels of HGF, that correlates with a negative prognosis and a high risk of mortality [89,90]. Risk prediction obtained with HGF levels analysis seems to have a prognostic value superior to conventional parameters [89,90]. HGF has been also identified as a prognostic marker of severity in patients with hypertension [91,92]. Finally, HGF levels were found elevated also in nonagenarian population with increased mortality [93]. Recently, HGF has been proposed as a precocious biomarker for the acute phase of the bowel inflammation [94]. HGF up-regulation is induced by the inflammatory mediators produced by infiltrated leucocytes in injured organs. Indeed, HGF production can be triggered by pro-inflammatory cytokines both in intra- and extra-hepatic tissues [95]. The increase of HGF in injured tissues is undoubtedly required for self-repair. However, after the resolution of the injury a response restoring the physiological level of HGF is necessary. It is, thus, possible that the maintenance of high levels of HGF could become detrimental and could have a role in the progression of the disease. In this line, it should be considered the hypothesis that elevated levels of HGF desensitize the system by down-regulating Met receptor [7]. The putative contribution of the HGF/Met axis in cardiac mortality has to be further investigated in the future. Overall these results suggest that Met ligand could be used as a predictor marker of severity and mortality for different diseases, among which those of the cardiovascular system.

## 11. Conclusions and Future Perspectives

**Figure 1:**
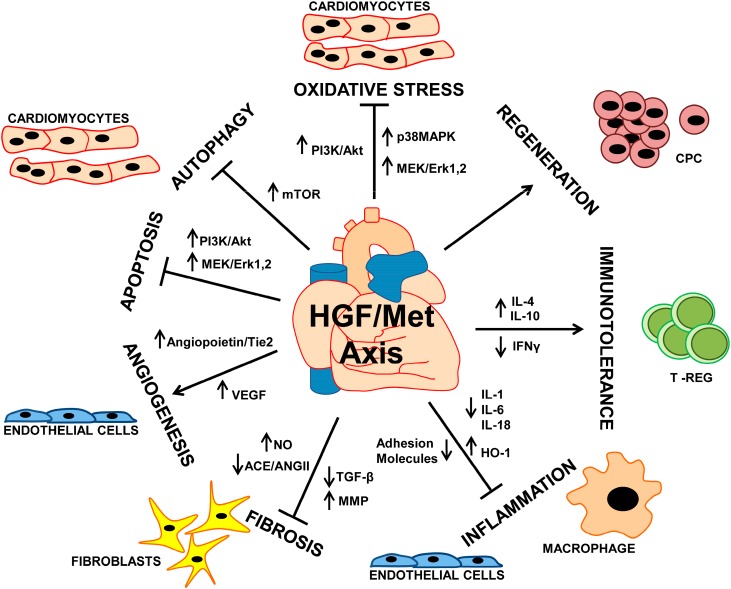
Cardioprotective functions of HGF/Met axis in the heart. Schematic representation of the cardioprotective roles played by HGF in the heart. HGF/Met axis acts on the different cell populations of the cardiac tissue. HGF exerts anti-oxidant, anti-autophagic and anti-apoptotic effects in cardiomyocytes. It has a mitogenic effect on endothelial cells, leading to induction of angiogenesis, and inhibits fibrosis. Furthermore, HGF/Met presents an anti-inflammatory action, through modification of endothelial cells and macrophages. It also promotes expansion of regulatory T cells (T-REG), leading to immune tolerance. Finally, HGF activates cardiac progenitor cells (CPC) to induce regeneration of injured heart.

HGF/Met axis controls heart homeostasis in the adults through cell autonomous effects in cardiomyocytes, which include protection from apoptosis and anti-oxidant function (Figure 1). The same biological processes are also stimulated by endogenous and exogenous HGF during heart repair following MI. Moreover, activation of HGF/Met axis after injury has cardioprotective effects through multiple paracrine actions on the different cell types populating the cardiac tissue (Figure 1). Further studies performed through conditional, lineage specific *Met* knockout models will be useful to better determine the physiological roles of HGF/Met axis in the various cell populations which constitute the heart tissues, such as cardiac fibroblasts, endothelial cells, CPCs, *etc*. These studies might reveal a more complex picture of HGF/Met involvement in the physiological regulation of cardiac functions and will provide a rational basis to follow the potential cardiotoxicity of Met-targeted anti-tumor therapies in selected cancer patients. On the other hand, the well-established cardioprotective effects of HGF in MI may be exploited in other cardiac diseases, such as heart failure, inherited cardiomyopathies, myocarditis and heart transplantation. Moreover, the HGF-mediated cardioprotection could be used to mitigate the cardiotoxicity elicited by anti-cancer drugs. Administration of biologically active HGF in patients may be limited by the complexity of the molecule and by the mechanism of its activation. Fortunately, a spectrum of HGF-based molecules and mimics of HGF have been already developed [23,96,97,98,99] and wait to be applied as a new arsenal for the therapy of cardiac diseases.
